# Dynamic beat-to-beat blood pressure estimation using a multi-modal wearable deep learning approach

**DOI:** 10.1088/1361-6579/ae85b4

**Published:** 2026-07-27

**Authors:** Qiao Li, Zichao Shen, Mohamed Almadi, Ye Yang, Ning Zhang, Jacqueline Gong, Gary Strangman, Ting Xiang, Yuanting Zhang, Gari D Clifford, Quan Zhang

**Affiliations:** 1 Department of Biomedical Informatics, Emory University, Atlanta, GA 30322 United States of America; 2 Department of Computing, Imperial College London, London, United Kingdom; 3 Neural Systems Group, Massachusetts General Hospital, Harvard Medical School, Charlestown, MA 02129, United States of America; 4 Department of Biomedical Technology, College of Applied Medical Sciences,King Saud University, Riyadh 11495, Saudi Arabia; 5 School of Engineering, Brown University, Providence, RI 02912, United States of America; 6 Department of Electronic Engineering, The Chinese University of Hong Kong, Hong Kong SAR, People’s Republic of China; 7 Hong Kong Institutes of Medical Engineering, Hong Kong Special Administrative Region of China, People’s Republic of China; 8 AICARE Bay Lab at Guangdong Medical University, Dongguan 523898, People’s Republic of China; 9 Department of Biomedical Engineering, Georgia Institute of Technology, Atlanta, GA 30332 United States of America

**Keywords:** dynamic blood pressure estimation, noninvasive blood pressure estimation, superficial temporal artery tonometry, multi-modality wearable signals, signal quality index, deep learning

## Abstract

*Objective.* Cuffless blood pressure (BP) monitoring technologies, primarily based on pulse transit time (PTT) or photoplethysmography (PPG), frequently suffer from calibration drift due to their reliance on blood volume surrogates rather than direct pressure measurement. To address this physiological limitation, this study presents a multi-modal deep learning framework that integrates superficial temporal artery tonometry (STAT), which captures high-fidelity pressure waveform morphology, with electrocardiography (ECG) and PPG signals. *Approach.* A custom wearable device was developed to simultaneously acquire these signals during dynamic perturbations. Extracted features included heart rate from ECG, PTT from ECG-PPG pairs, and BP-related metrics derived from PTT and STAT. Signal quality indices for ECG, PPG, and STAT signals were also computed to assess signal reliability. A temporal convolutional network (TCN) model was designed to capture the complex, non-linear dependencies between these multi-modal features and beat-to-beat BP. *Main results.* The approach was rigorously validated using leave-one-subject-out cross-validation on 29 recordings from ten healthy volunteers undergoing isometric handgrip exercises. The proposed TCN model significantly outperformed traditional PTT and STAT-only baselines, achieving a mean absolute difference of 5.58 mmHg for systolic BP , 4.39 mmHg for mean BP and 4.34 mmHg for diastolic BP. Notably, the TCN model exhibited significantly lower errors (*p* $<$ 0.05, Wilcoxon Test) during dynamic BP fluctuations compared to baseline models. *Significance.* The study demonstrates that fusing tonometry-derived pressure morphology with hemodynamic timing features effectively mitigates the limitations of conventional PTT methods. This approach offers a robust solution for continuous, calibration-resilient BP estimation.

## 1. Introduction

Continuous blood pressure (BP) is one of the most critical monitoring parameters during anesthesia, surgery, and critical care. The current gold standard for continuous BP technology, arterial line (a-line) BP monitoring, involves placing a catheter pressure sensor into an artery to measure BP. However, the disadvantages of the invasive BP technology, such as physical pains, risk of infection, high cost, and requirement of trained personnel, restrict its use in perioperative continuous BP monitoring.

Extensive effort has been made in both academia and industry to develop noninvasive continuous BP technologies, yet the common major challenge is still stability (or drift). Among these attempts, devices based on Peñáz’s technology (volume-clamp, Finapres) require frequent calibration (e.g. every 10 min) to correct for measurement drift; they are also limited by the discomfort and degradation of signal quality (from long periods of squeezing on the finger), large device size, and high cost (Chung *et al* 2013). The latest progress in ultrasound-based noninvasive BP monitoring has shown significant potential in continuous BP monitoring (Zhou *et al* 2025), however, its design of a sensor film attached to the neck makes it susceptible to drift from the deformation and position changes of the blood vessel and soft tissue. Other cuffless BP approaches, such as those using pulse transit time (PTT) (Wang *et al* 2018), tonometry (Pressman and Newgard 1963, Lehmann 2000), and hemodynamics-based technologies (McCombie *et al* 2007), have long been analyzed as potential candidates for continuous BP monitoring. However, research by other groups and us has shown that the relationship between PTT and BP is not yet fully developed, as it is not sensitive to rapid BP fluctuations. Moreover, PTT-based approaches face challenges regarding accuracy and calibration (Buxi *et al* 2015). For example, factors adversely affecting PTT calibration include motion artifacts, changes in the contact force of sensors, temperature, and vascular muscle tone (Sharma *et al* 2017).

Along with the rapid evolution of deep learning techniques, more studies are focusing on leveraging DL algorithms for non-invasive BP estimation using photoplethysmography (PPG) waveform, such as convolutional neural networks CNNs, (Baek *et al* 2019), long short-term memory (LSTM) and gated recurrent units (GRUs) (El-Hajj and Kyriacou 2021), deep convolutional autoencoders DCAEs, (Sadrawi *et al* 2020), U-Net (Ibtehaz *et al* 2020) and V-Net architecture (Hill *et al* 2021), U-Net with self-attention (Kim *et al* 2022) and transformer architecture (Ma *et al* 2024). By training on large-scale datasets of PPG waveforms synchronized with corresponding BP measurements, DL models can learn intricate relationships between the waveform characteristics and BP values. This approach holds significant potential for personalized BP estimation, as it can adapt to individual physiological variations and dynamic changes over time. However, the DL models face some challenges like long training time, substantial computational resources and overfitting issues.

Although good noninvasive BP estimation numbers have been reported in the literature, most studies have focused on static BP without significant BP fluctuations. To implement dynamic BP estimation, (Ding *et al* 2017) introduced various maneuvers with induced BP changes on 33 subjects with and without hypertension. They extended the PTT-based method by introducing a new indicator, the PPG intensity ratio. The results showed that the BP estimation errors increased significantly under dynamic maneuvers and over extended calibration intervals. More recently, (Xiang *et al* 2024, 2025) introduced a multi-modal physiological model and a deep learning model incorporating cold pressor tests in 23 subjects. Using electrocardiography (ECG), PPG, impedance plethysmography, and skin temperature signals, their results demonstrated that multi-modal fusion significantly outperformed single-modality approaches in dynamic BP estimation.

A recent critical review by Mukkamala *et al* (2025), Wehbe and Hiremath (2026)highlight a sobering reality: most current devices, predominantly based on PTT or pulse wave analysis of PPG signals, fail to maintain accuracy beyond a narrow calibration window. A fundamental limitation, as noted by Dias *et al* (2025), is that PPG measures blood volume changes—a surrogate distinct from pressure. Consequently, these methods often struggle to distinguish between true BP variations and changes in vasomotor tone, leading to significant drift and reliability issues in dynamic settings.

**Figure 1.**
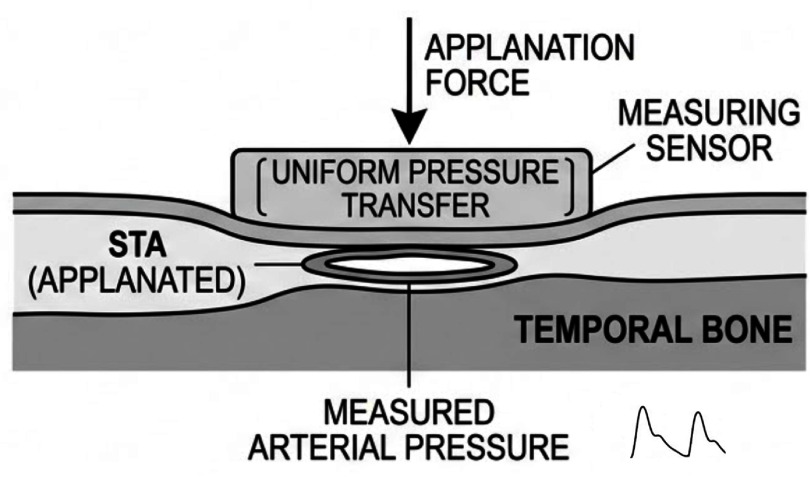
The physiological principle of STAT.

To overcome the intrinsic limitations of volume-based sensing, we propose integrating superficial temporal artery (STA) tonometry (STAT) into a multi-modal wearable framework (Canning *et al* 2016, Luo *et al* 2018, Zhang *et al* 2018, Zhang and Zhang 2023, Zhu *et al* 2026). The physiological and mechanical principle of STAT is illustrated in figure 1. Based on the principles of applanation tonometry, when a superficial artery is partially flattened against a rigid anatomical backing—in this case, the temporal bone—the circumferential wall tension of the vessel becomes parallel to the sensor surface. Consequently, the tangential forces within the arterial wall are effectively canceled out, allowing the external force transducer to directly measure the continuous transmural pressure waveform. Because the STA is highly accessible and directly supported by the smooth surface of the cranium, STAT provides a high-fidelity, beat-to-beat representation of the intra-arterial pressure dynamics without the confounding effects of deep tissue attenuation. Unlike PPG, arterial tonometry applies applanation force to flatten the vessel, yielding a waveform that physically approximates intra-arterial pressure (Wang *et al* 2018). Although traditional tonometry requires cumbersome manual manipulation, our custom wearable STAT sensor enables continuous acquisition of high-fidelity pressure morphology from the temporal artery. We hypothesize that fusing this direct pressure-related information (STAT) with conventional hemodynamic markers (ECG and PPG) can robustly compensate for the vasomotor-induced errors that plague PTT-only models. Compared with traditional tonometry performed on other arteries, such as radial artery at the wrist (Saugel *et al* 2013, Greiwe *et al* 2018) or finger arteries used in BP measurement (Chandrasekhar *et al* 2018), STA appears to be an ideal location for continuous, noninvasive BP monitoring due to its ease of localization, support from the underlying bone structure that facilitates compression, and ability to provide complete waveform data with a bandwidth exceeding 20 Hz. Traditional tonometry requires a ‘proper hold-down pressure’ that flattens but does not occlude the target artery (applanation) (Pressman and Newgard 1963, Lehmann 2000). However, the tonometry-based BP technique was susceptible to fluctuations in hold-down pressure, resulting in compromised stability in BP monitoring.

The physiological advantage of STAT lies in its measurement principle: applanation tonometry directly flattens the artery against a rigid structure to measure the transmural pressure pulse. Unlike PTT, which serves as a surrogate metric for arterial stiffness and is susceptible to confounding factors such as vascular compliance and vasomotor tone, STAT provides a high-fidelity morphological representation of the intra-arterial pressure. By capturing pressure-specific features (e.g. peak intensity and waveform area), STAT complements the timing-based information of PTT, allowing the model to capture acute, dynamic pressure fluctuations that timing-based features inherently overlook.

To overcome the effect of hold-down pressure drift on BP estimation, several of the vital signs used routinely in patient monitoring, such as ECG and PPG, which contain rich BP information, were introduced for data fusion. However, effectively fusing these heterogeneous signals (mechanical STAT, optical PPG, and electrical ECG) presents a complex modeling challenge. The relationship between these multi-modal features and BP is highly non-linear and dynamic, rendering traditional regression techniques inadequate. To address this, we developed a temporal convolutional network (TCN) designed to capture long-range temporal dependencies and morphological subtleties across modalities. Crucially, (Mukkamala *et al* 2025) warned that many machine learning-based BP studies suffer from ‘data leakage,’ where models are trained and tested on the same subject, leading to over-optimistic results that fail to generalize. To ensure the rigor and physiological validity of our approach, we employed leave-one-subject-out cross-validation (LOSOCV) strictly to validate our approach on a dataset containing dynamic BP fluctuations induced by isometric handgrip exercises. This study demonstrates that extracting features from a tonometry-based multi-modal system significantly outperforms traditional PTT approaches, offering a viable path toward calibration-resilient BP monitoring. In this study, we proposed a multi-modality signal fusion and a deep learning approach to improve the stability of noninvasive continuous BP monitoring. A signal quality index (SQI) driven Kalman filter (KF) approach (Li *et al* 2008, 2009) was also introduced to overcome the hold-down pressure drift. BP information from synchronously collected STAT and the extracted PTT from ECG and PPG were fused by a TCN, accompanied by the SQI of the signals as the model’s inputs.

## 2. Methodology

### 2.1. Dataset

We collected 29 records from ten healthy volunteers using our multi-modality data collection system during 2017 and 2018 (Jiang *et al* 2020). The average length of the records is 37.24 $\pm$ 10.06 min. In order to introduce dynamic BP fluctuation, sustained handgrip exercise was used during each data collection. The data collection procedure involves approximately 20 min of rest, followed by three to four separate sessions of handgrip exercise, each with a 10 min rest period. Each record includes synchronous continuous STAT, ECG, and PPG signals. Crucially, to prevent cumulative clock discrepancies and temporal drift that could compromise precision timing features such as PTT, all physiological signals (STAT, ECG, and PPG) were strictly co-registered and acquired synchronously. The multimodal data streams were digitized at 250 Hz using a unified, custom-designed hardware platform with a single master crystal clock. This hardware-level synchronization ensures zero temporal discrepancy or phase misalignment between the electrical, optical, and mechanical modalities across the entire duration of the recording periods, consistent with our established system design (Jiang *et al* 2020). The continuous BP signal from the Finapres device was recorded synchronously as the reference.

### 2.2. Preprocessing

To ensure the framework is fully compatible with real-time wearable deployment while eliminating non-causal operations, all preprocessing steps were restricted to causal, linear-phase finite-impulse-response (FIR) filters. The ECG signal was preprocessed using a FIR notch filter to eliminate power-line noise. A state-of-the-art QRS detector (jqrs) was used for ECG R-peak detection (Johnson *et al* 2015). The detector consists of a window-based peak energy detector, which is extremely robust to noise. An ECG SQI (bSQI) was extracted to assess the signal quality (Li *et al* 2008). The bSQI quantifies the agreement between two independent QRS detection algorithms based on different approaches (e.g. jqrs and wqrs (Zong *et al* 2003)). It is calculated as the ratio of synchronously detected peaks to the total number of peaks detected by either algorithm. Correspondingly, the STAT signal was filtered by an FIR low-pass filter with a cut-off frequency of 5 Hz. Crucially, both filters were mathematically constrained to have an identical group delay. This matched-group-delay configuration guarantees that the relative temporal relationships—specifically the interval between the ECG R-peak and the STAT fiducial point for PTT calculation—are perfectly preserved without phase distortion or cross-modal temporal misalignment. The resulting system introduces only a fixed, deterministic processing latency equal to the group delay, which is negligible for continuous hemodynamic monitoring. A state-of-the-art beat detection algorithm for pulsatile signals such as BP or PPG was used for STAT beat detection (Li and Clifford 2012).

### 2.3. Signal quality of STAT

To ensure the reliability of the TCN model, a multi-stage SQI framework was implemented for the STAT signal. Two independent SQI metrics were calculated and combined to generate a final quality score ($\mathrm{SQI}_\mathrm{final}$) for every detected beat. (1) Morphological SQI ($\mathrm{SQI}_\mathrm{morph}$): Following the DTW-based template matching approach, a dynamic beat template was maintained for each signal. For every newly detected beat, the similarity factor was calculated by matching the individual beat waveform to the dynamic template using DTW to account for slight physiological variations in pulse shape. A higher correlation indicates greater morphological fidelity and less interference from motion artifacts. (2) Timing consistency SQI ($\mathrm{SQI}_\mathrm{time}$): This multi-modality metric leverages the inherent temporal relationship between the electrical activity (ECG) and the mechanical pressure pulse (STAT). Given the high SNR typically observed in our ECG recordings, $\mathrm{SQI}_\mathrm{time}$ was defined as the agreement between the R-peak detection and the subsequent STAT beat detection. A match was confirmed if a STAT pulse was detected within a physiologically plausible window following the QRS complex. The final quality score was computed as the product of the two indices:

\begin{equation*} \mathrm{SQI}_\mathrm{final} = \mathrm{SQI}_\mathrm{morph} \times \mathrm{SQI}_\mathrm{time}. \end{equation*}

To ensure reliable BP dynamics, an SQI threshold of 0.7 was selected empirically based on preliminary experiments. This strict criterion ensured that only pulses with both high morphological integrity and valid physiological timing were used for BP estimation. Data segments with SQI values below this threshold were excluded from the analysis to eliminate the effects of movement artifacts, which could compromise BP estimation accuracy. The proposed SQI algorithms were applied to the PPG signal as well.

### 2.4. Feature extraction

To estimate systolic BP (SBP) and diastolic BP (DBP) from the STAT signal, we calibrated the STAT signal only once using the Finapres reading at the start of the data collection. The beat-by-beat SBP and DBP were extracted based on the STAT beat detection. We developed a method to address the hold-down pressure drift, which can significantly affect BP accuracy. We introduced a median filter to track and correct this drift. The filter was designed with a window length corresponding to five minutes of data, which is sufficient to capture the slow, gradual nature of the hold-down pressure drift. The filtered SBP and DBP were then inputted into an SQI-driven KF for a robust BP estimation. The detail of the SQI-driven KF can be referred to Li *et al* (2009). This approach of median filtering combined with SQI-driven KF and SQI-based quality control offers a robust method for mitigating hold-down pressure drift, leading to more accurate and reliable BP estimations. In parallel, PTT was extracted from synchronous ECG and PPG signals, and BP estimation was performed using two state-of-the-art PTT-based algorithms (Ding *et al* 2017). Due to varying definitions in the literature, we explicitly define PTT in this study as the time interval between the ECG R-peak and the maximum slope point (maximum first derivative) of the subsequent PPG rising edge. Although this interval is technically referred to as pulse arrival time as it includes the cardiac pre-ejection period , it serves as a reliable surrogate for arterial stiffness in beat-to-beat BP estimation. The maximum slope point was selected as the fiducial marker due to its superior stability against physiological noise and baseline fluctuations. Based on this PTT definition, two established physiological mapping models were implemented as baseline features:

\begin{equation*}\mathrm{PTT}_1 : \begin{cases} \mathrm{DBP} = \mathrm{DBP}_0 - \frac{2}{\gamma \mathrm{PTT}_0}\left(\mathrm{PTT} - \mathrm{PTT}_0\right) \\ \mathrm{SBP} = \mathrm{SBP}_0 - \frac{2}{\gamma \mathrm{PTT}_0}\left(\mathrm{PTT} - \mathrm{PTT}_0\right) \end{cases}\end{equation*}

\begin{equation*}\mathrm{PTT}_2 : \begin{cases} \mathrm{DBP} = \mathrm{MBP}_0 + \frac{2}{\gamma} \ln \frac{\mathrm{PTT}_0}{\mathrm{PTT}} - \frac{1}{3} \mathrm{PP}_0 \cdot \left( \frac{\mathrm{PTT}_0}{\mathrm{PTT}} \right)^2 \\ \mathrm{SBP} = \mathrm{DBP} + \mathrm{PP}_0 \cdot \left( \frac{\mathrm{PTT}_0}{\mathrm{PTT}} \right)^2 \end{cases}\end{equation*}

where $\mathrm{SBP}_0$ and $\mathrm{DBP}_0$ are initial calibration values, $\mathrm{PTT}_0$ is the reference transit time, and $\gamma$ is a subject-specific vascular parameter.

A multi-dimensional feature set was extracted for each detected beat to serve as the input to the TCN model, as summarized in table 1. To capture both the direct pressure information and the underlying hemodynamic trends, we included morphological metrics derived from STAT (e.g. mean BP (MBP) and pulse pressure) alongside timing-based surrogates (Raw PTT and PTT-based BP estimates using two distinct formulas). Furthermore, heart rate (HR) and real-time SQI vectors for each signal modality were incorporated. This configuration allows the network to adaptively gate the contributions of different sensors, prioritizing high-fidelity STAT segments for dynamic changes while relying on PTT and HR for baseline stability during periods of motion or sensor coupling variations.

**Table 1.** Detailed input feature set for the TCN-based BP estimation model.

Category	Feature names	Description & physiological significance
STAT-derived	SBP$ _\mathrm{STAT}$, DBP$ _\mathrm{STAT}$	Beat-by-beat BP values derived from tonometry waveform
Pressure	MBP$ _\mathrm{STAT}$	Mean arterial pressure from the STAT morphology
	PP$ _\mathrm{STAT}$	Pulse pressure representing the amplitude of pressure swing
	Norm-SBP, Norm-DBP	Normalized BP metrics to capture relative morphological shifts
PTT-derived	SBP$ _\mathrm{PTT1}$, DBP$ _\mathrm{PTT1}$	BP estimated via PTT1 model
Timing	SBP$ _\mathrm{PTT2}$, DBP$ _\mathrm{PTT2}$	BP estimated via PTT2 model
	Raw PTT	Direct interval between ECG R-peak and pulse arrival
Contextual &	HR$ _\mathrm{ECG}$	Heart rate reflecting autonomic cardiovascular state
Quality	STAT SQI	Signal quality index for the tonometry modality
	PPG SQI	Signal quality index for the optical modality
	ECG SQI	Signal quality index for the electrical modality

### 2.5. TCN

**Figure 2.**
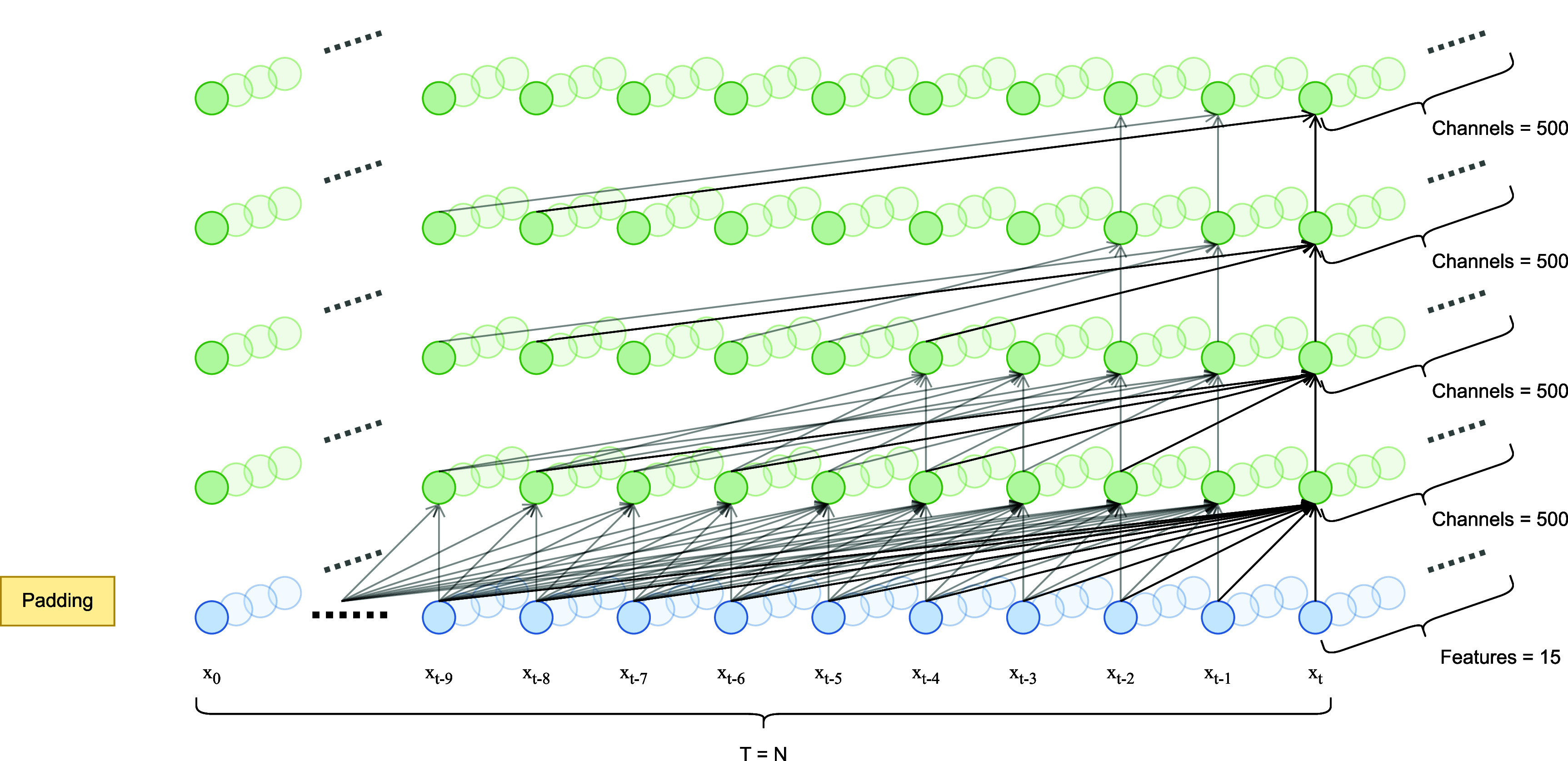
Structure of the proposed TCN model for dynamic beat-to-beat BP estimation.

**Figure 3.**
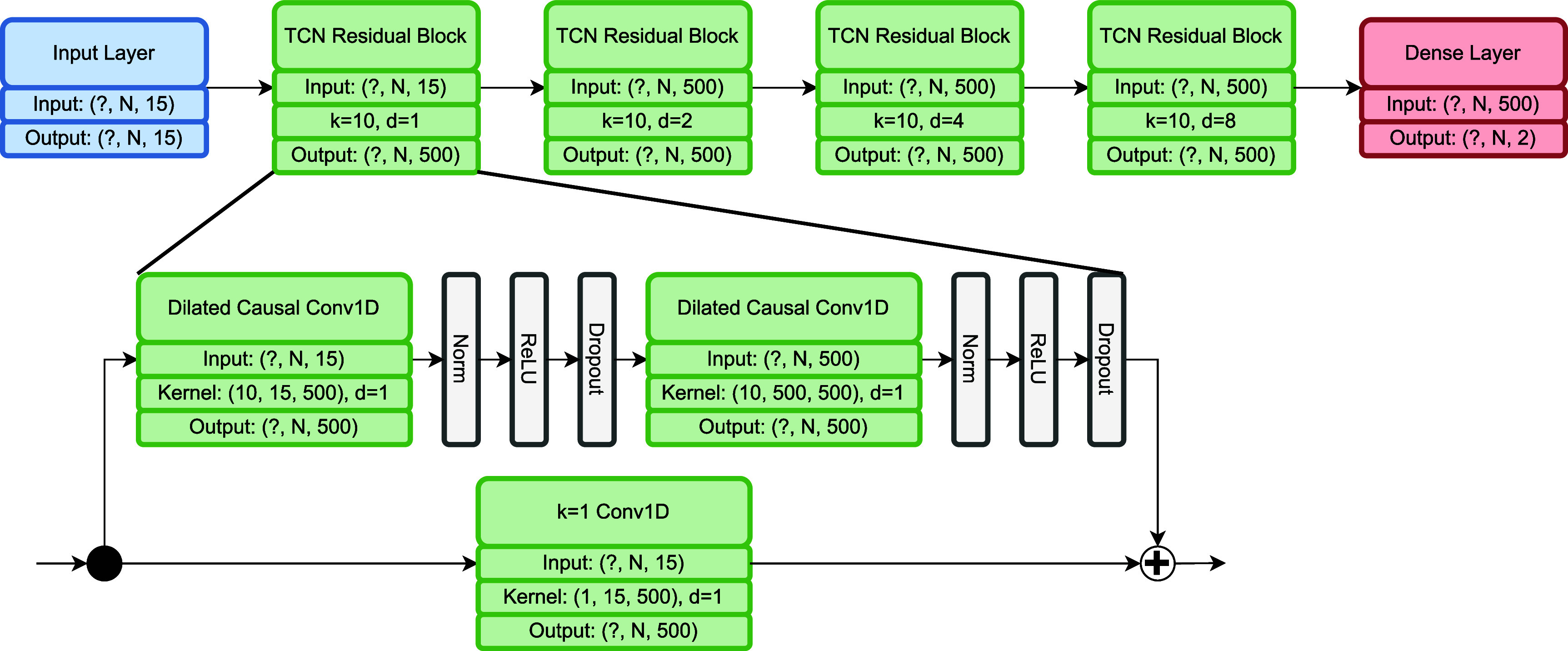
TCN architecture and detailed parameters for dynamic beat-to-beat BP estimation.

To fuse multi-modal signals for BP estimation, we developed a TCN approach (Bai *et al* 2018) designed to track dynamic, beat-by-beat systolic and DBP. The TCN model offers several distinct advantages: (1) Causal convolutions: The architecture is causal, ensuring that the output at *time t* only depends on inputs from *time t* and earlier, making it ideal for real-time BP estimation; (2) Flexible input length: TCNs can handle sequences of any length and map them to an output sequence of the same length through zero-padding, similar to recurrent neural networks but without the complexity of vanishing gradients; (3) Dilated Convolutions: The model employs dilated convolutions, where a dilation factor d is introduced at different layers. This enables the TCN to capture long-range BP dependencies more effectively than conventional methods. These features make the TCN not only more accurate but also simpler and more efficient than traditional recurrent networks like LSTMs and GRUs. In our model, the input signals include BP estimations from STAT, the SQI of STAT, BP estimations from PTT, the SQIs of ECG and PPG, HR, and the PTT signal itself. The synchronous BP from the Finapres system is used as the reference. The TCN structure and its parameters are illustrated in figures 2 and 3, demonstrating the robustness and clarity of the model in handling complex multi-modal data for dynamic BP estimation. While the base TCN architecture is well-established, the specific innovation in this framework is its multi-modal fusion strategy optimized for cross-modality drift compensation. Traditional methods typically rely on rigid mathematical filters to mitigate baseline drift, which often suppresses physiological variations. In contrast, our TCN-based framework learns a non-linear mapping between direct pressure dynamics (STAT) and surrogate hemodynamic timing features (PTT). By concatenating real-time SQI feature vectors at the input stage, the network implements an implicit attention mechanism. It dynamically adjusts the receptive field’s sensitivity, effectively leveraging PTT for long-term baseline stability while relying on STAT’s high-fidelity fluctuations to capture rapid, transient pressure changes. The use of dilated causal convolutions specifically enables the model to capture long-range dependencies in PTT (for baseline) while maintaining the fine-grained temporal resolution needed for STAT dynamic.

### 2.6. Validation and statistics

We used a LOSOCV approach to train, validate and test the performance of the proposed model. N minus 1 subjects were used as the training fold, and 1 subject was left as the test fold. The leave-one-subject-out procedure was repeated *N* times with each of the *N* subjects tested once and the results were averaged or accumulated. During each training loop, the *N*-1 subjects were further split into training and validation sets with *N*-2 subjects in the training set and 1 subject in the validation set. The model that achieved the best performance on the validation set was selected for testing on the remaining subject. The distribution of error residuals was first assessed using the Shapiro–Wilk test. Given the significant deviation from normality ($p < 0.05$), the Wilcoxon signed-rank test was employed as a non-parametric alternative to evaluate the statistical significance ($p < 0.05$) between the TCN and baseline models. To quantify the uncertainty of the performance metrics without making distributional assumptions, 95% confidence intervals (CIs) were calculated using a non-parametric Bootstrap method with 1000 resamples.

## 3. Results

**Figure 4.**
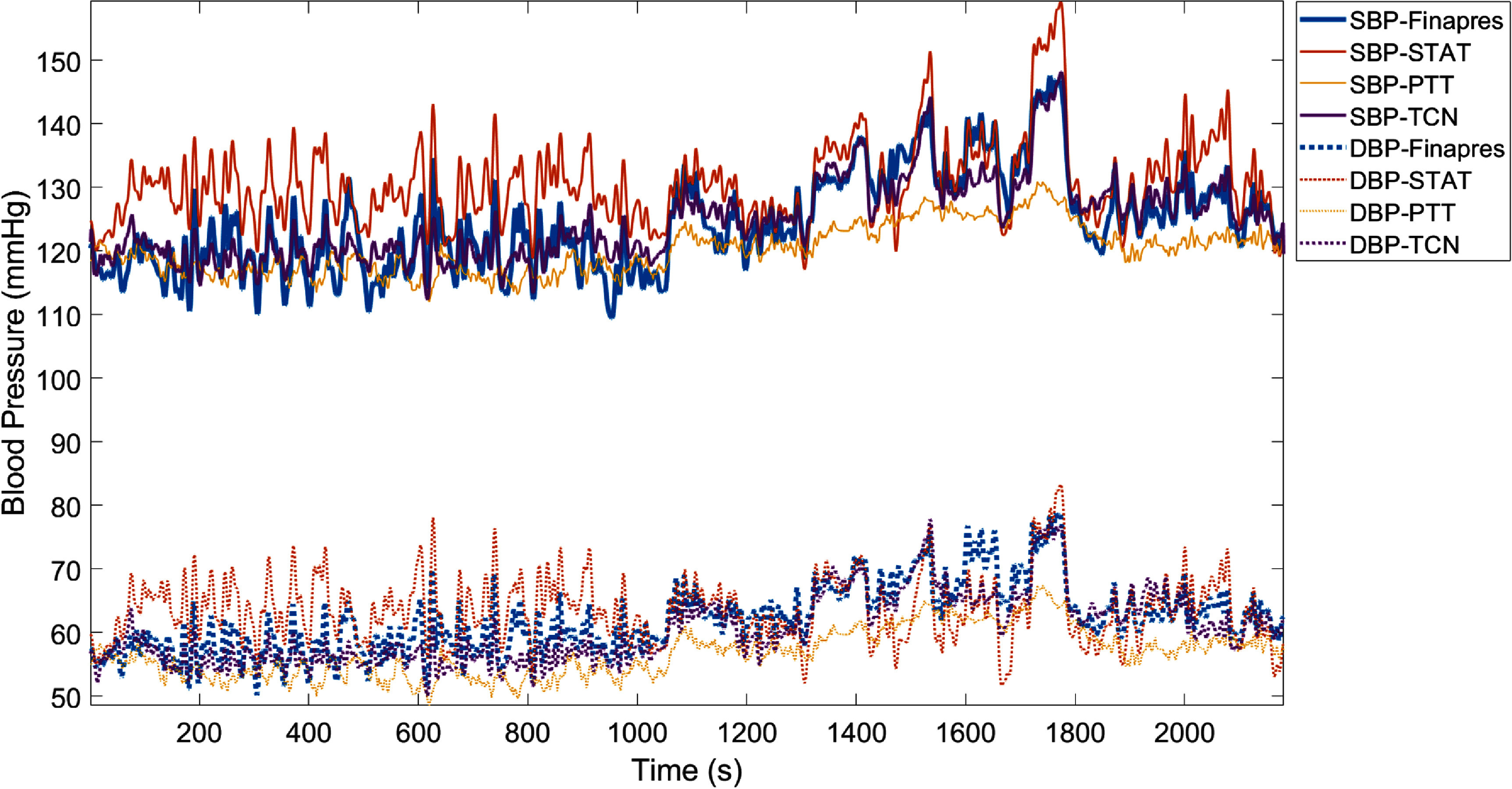
An example of the BP estimations from algorithms and the BP from Finapres reference.

**Figure 5.**
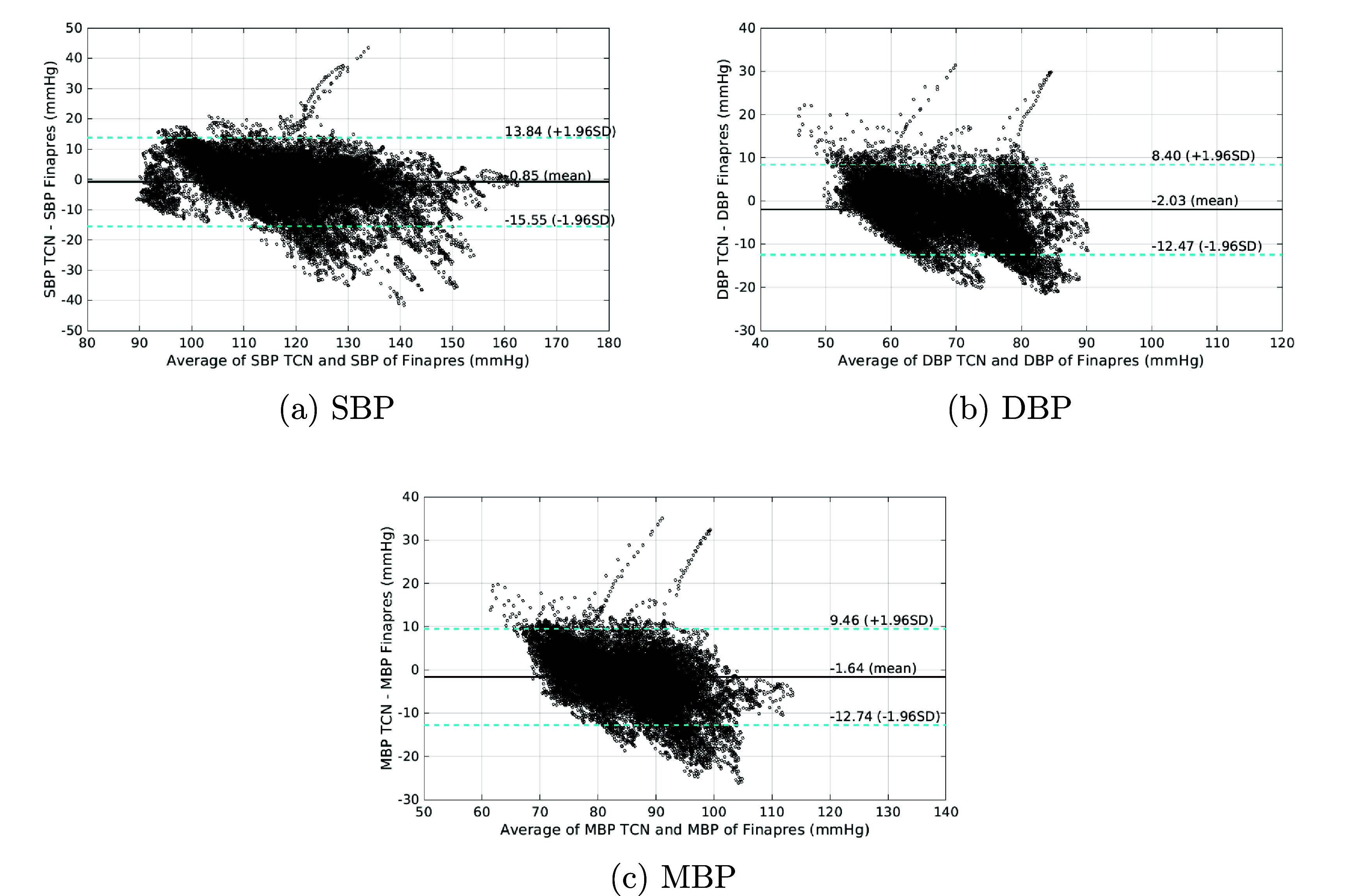
Bland–Altman plots between the predictions of the TCN algorithm and the references for (a) SBP, (b) DBP, and (c) MBP.

**Figure 6.**
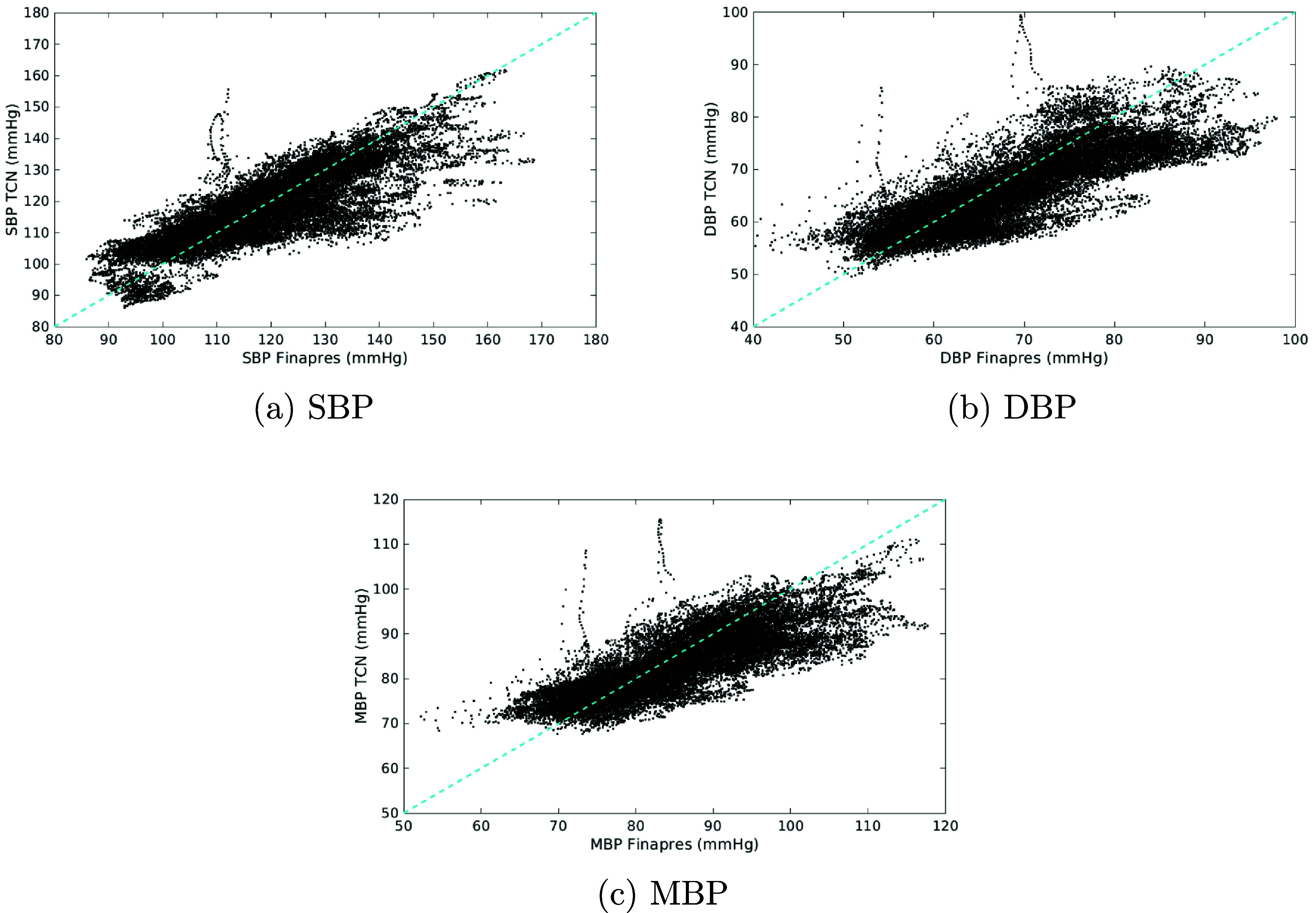
Scatter plots between the predictions of the TCN algorithm and the references for (a) SBP, (b) DBP, and (c) MBP.

The mean absolute difference (MAD) between BP estimations from the proposed TCN algorithm and the Finapres reference, as well as the MAD for BP estimations from the STAT and PTT methods, are presented in table 2. The LOSOCV results demonstrate that the TCN model outperformed the other methods, achieving a MAD of 5.58 mmHg for SBP, 4.39 mmHg for MBP, and 4.34 mmHg for DBP-falling below the 6 mmHg (for SBP) and 5 mmHg thresholds (for DBP and MBP), respectively. Figure 4 provides an example of the BP estimations from the algorithms alongside the Finapres reference. Compared to the STAT-only method and the PTT-based method, the TCN accurately tracks the dynamic BP fluctuations. Additionally, the Bland–Altman plots for MBP, SBP and DBP, comparing the predictions of the TCN algorithm to the reference, are displayed in figure 5. The scatter plots of the MBP, SBP and DBP between the prediction of the TCN algorithm and the reference are shown in figure 6.

**Table 2.** LOSOCV validation result of BP estimation vs Reference (DR: drift removal).

	Estimation error in mmHg (MAD)		
BP Estimation method	SBP [95% CI]	DBP [95% CI]	MBP [95% CI]
STAT-based model before DR	10.11 [10.01, 10.20]	4.99 [4.94, 5.04]	6.13 [6.07, 6.19]
STAT-based model after DR	7.31 [7.24, 7.38]	4.88 [4.83, 4.92]	5.44 [5.39, 5.49]
PTT-based model	6.54 [6.47, 6.61]	4.83 [4.79, 4.87]	5.08 [5.04, 5.13]
TCN model	5.58 [5.52, 5.63]	4.34 [4.30, 4.37]	4.39 [4.35, 4.43]

**Figure 7.**
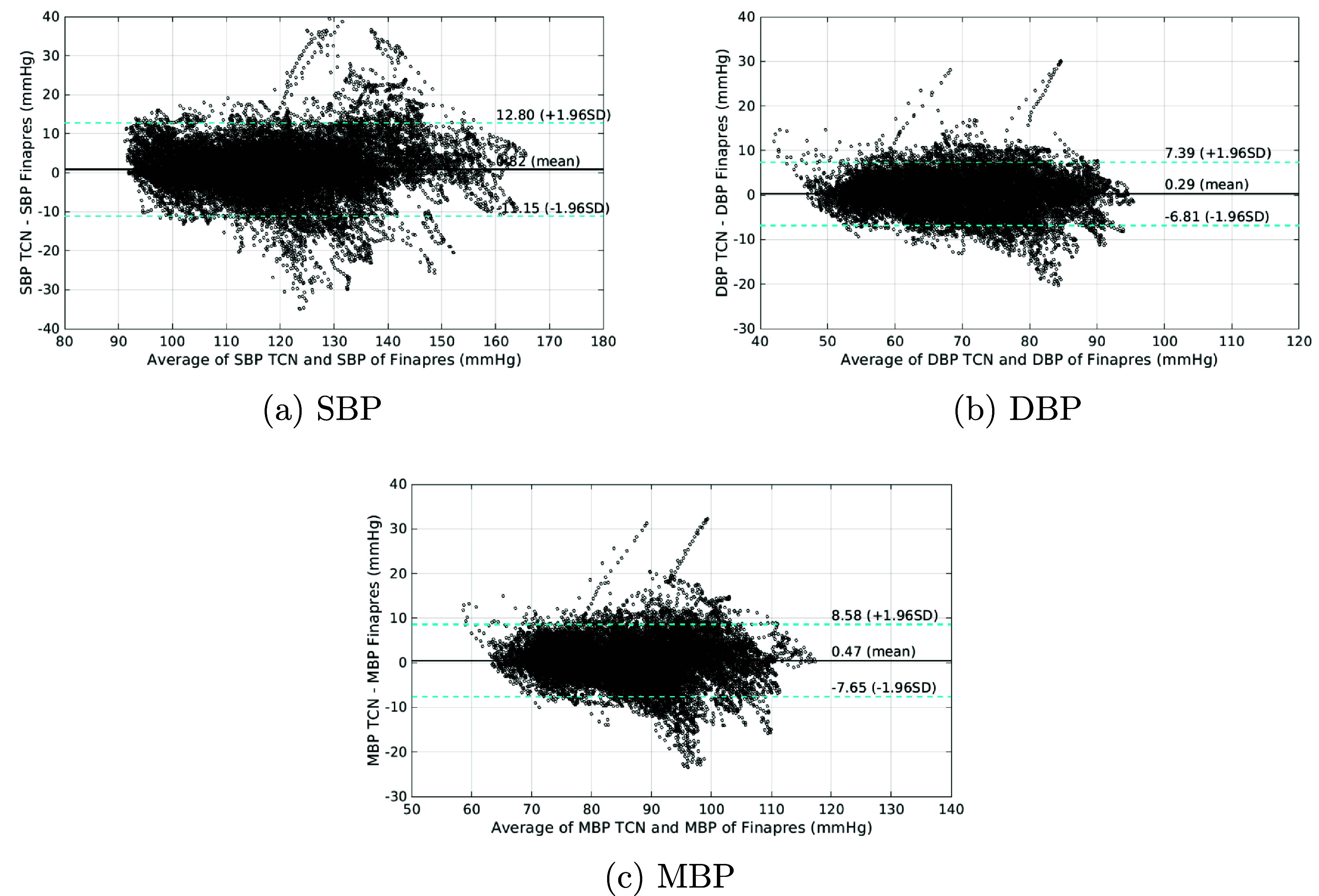
Bland–Altman plots with calibration points added every 10 min between the predictions of the TCN algorithm and the references for (a) SBP, (b) DBP, and (c) MBP.

**Figure 8.**
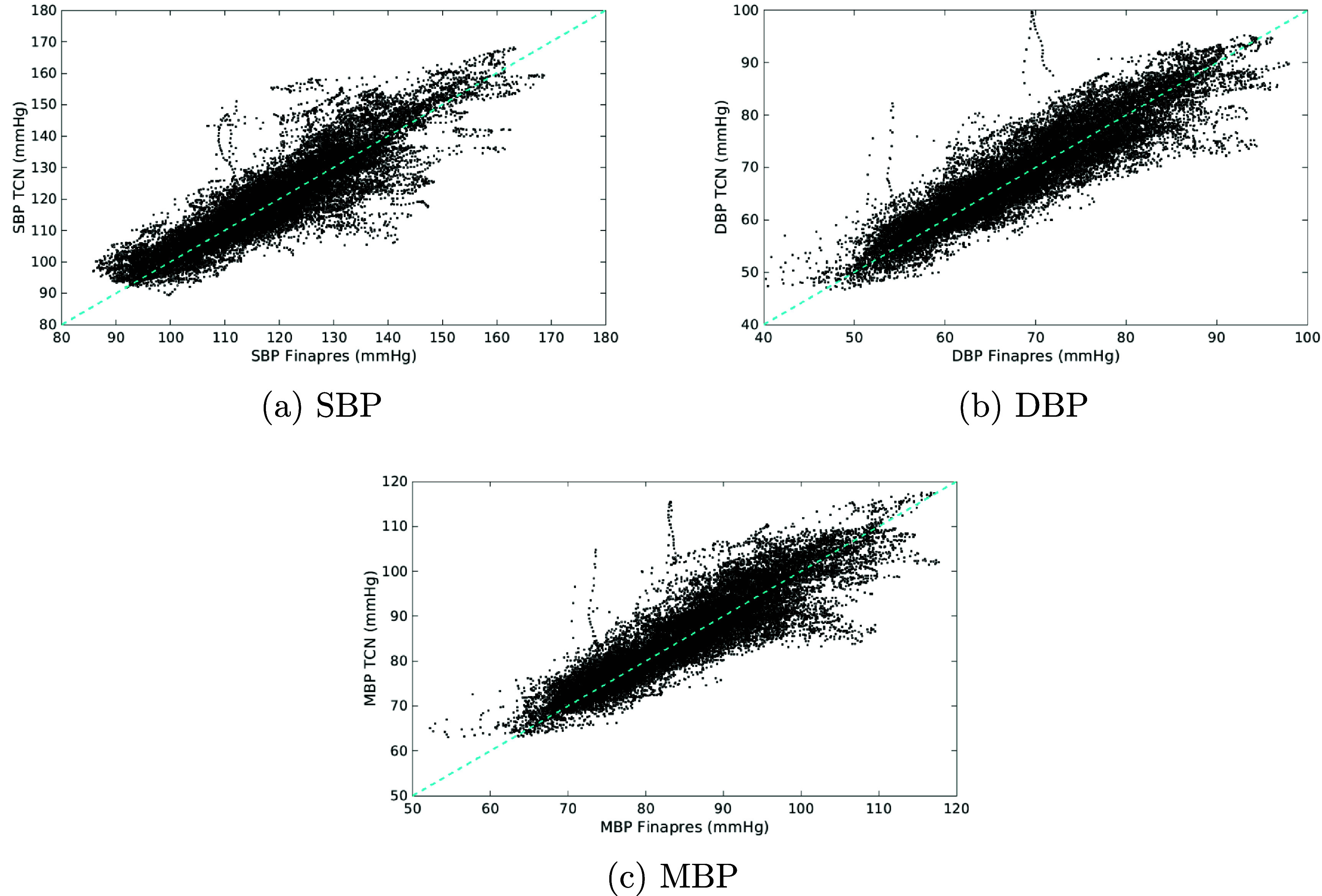
Scatter plots with calibration points added every 10 min between the predictions of the TCN algorithm and the references for (a) SBP, (b) DBP, and (c) MBP.

To investigate the effect of calibration on BP estimation, we introduced calibration points every 20 min and every 10 min. This approach involved recalibrating the STAT and PTT-based BP estimations according to the Finapres values at these intervals. The results of this calibration-based approach are shown in table 3, and the Bland–Altman plots for MBP, SBP and DBP with 10 min calibration intervals are shown in figure 7. The scatter plots of the MBP, SBP and DBP with 10 min calibration intervals are shown in figure 8. This analysis highlights the improvement in BP estimation with regular calibration.

**Table 3.** LOSOCV validation result with adding calibration points of BP estimation vs Reference.

		Estimation error in mmHg (MAD)		
Calibration interval	BP Estimation method	SBP [95% CI]	DBP [95% CI]	MBP [95% CI]
20 (min)	STAT-based model after DR	6.56 [6.50, 6.62]	4.63 [4.58, 4.68]	5.12 [5.07, 5.18]
	PTT-based model	5.44 [5.38, 5.49]	3.97 [3.93, 4.00]	4.22 [4.18, 4.26]
	TCN model	5.19 [5.14, 5.23]	3.08 [3.05, 3.11]	3.54 [3.51, 3.57]

10 (min)	STAT-based model after DR	5.45 [5.38, 5.52]	3.93 [3.88, 3.99]	4.24 [4.19, 4.30]
	PTT-based model	4.72 [4.67, 4.77]	3.43 [3.40, 3.47]	3.69 [3.65, 3.73]
	TCN model	4.41 [4.37, 4.46]	2.68 [2.65, 2.71]	3.01 [2.98, 3.04]

To evaluate the model’s performance in capturing dynamic BP changes, we divided the collected data into two parts: a stable segment and a dynamic segment. The stable segment covers the period from the start of data collection up until approximately two minutes before the handgrip exercise, where BP remains relatively steady. The dynamic segment begins two minutes before the handgrip and continues until the end of the handgrip session, where BP fluctuations are more pronounced. This segmentation allows us to assess the TCN model’s ability to handle both steady-state and fluctuating BP conditions. As shown in table 4, during the stable segment, the TCN model performed exceptionally well, achieving MAD values of 3.91 mmHg for SBP, 2.92 mmHg for MBP, and 2.90 mmHg for DBP—all were under 4 mmHg. More importantly, during the dynamic segment—where BP fluctuations are more significant—the TCN model continued to deliver strong results, with a MAD of 7.12 mmHg for SBP, 4.74 mmHg for MBP, and 3.91 mmHg for DBP. These results demonstrate the TCN model’s robustness, particularly in dynamic conditions where other models typically struggle. TCN’s ability to maintain reliable performance during both stable and dynamic phases underscores its superiority in tracking real-time BP changes under varying conditions.

**Table 4.** LOSOCV validation result of stable segment and dynamic segment.

		Estimation error in mmHg (MAD)		
Segments	BP Estimation method	SBP [95% CI]	DBP [95% CI]	MBP [95% CI]
Stable segment	STAT-based model after DR	5.03 [4.96, 5.09]	4.05 [3.99, 4.10]	4.11 [4.06, 4.17]
	PTT-based model	4.04 [3.99, 4.09]	3.59 [3.55, 3.63]	3.76 [3.72, 3.80]
	TCN model	3.91 [3.85, 3.96]	2.90 [2.86, 2.93]	2.92 [2.89, 2.96]

Dynamic segment	STAT-based model after DR	9.52 [9.38, 9.65]	5.62 [5.53, 5.70]	6.82 [6.72, 6.92]
	PTT-based model	8.57 [8.42, 8.72]	5.07 [4.99, 5.16]	5.96 [5.86, 6.06]
	TCN model	7.12 [7.01, 7.23]	3.91 [3.85, 3.97]	4.74 [4.67, 4.81]

## 4. Discussion and conclusions

The proposed TCN-based multi-modality dynamic BP estimation algorithm demonstrated superior performance compared to other methods. While the STAT signal is capable of providing a high dynamic response to BP fluctuations, it suffers from significant hold-down pressure drift, leading to BP estimation errors exceeding 10 mmHg before drift correction. Our proposed drift removal technique effectively mitigated this issue, reducing the MAD to 7.31 mmHg for SBP and 4.88 mmHg for DBP. However, even with these improvements, the SBP results still fall short of the IEEE standards for wearable, cuffless BP measuring devices (IEEE Standards Association 2014, 2019).

On the other hand, the PTT-based BP estimation algorithm achieved a MAD of 6.54 mmHg for SBP and 4.83 mmHg for DBP, but it struggled to track BP fluctuations dynamically. In contrast, the TCN-based algorithm achieved a MAD of under 5 mmHg for DBP (Grade A) and under 6 mmHg for SBP (Grade B), while maintaining a superior dynamic response to BP swings. This highlights the TCN model’s ability to capture both static and fluctuating BP changes more effectively than other algorithms.

To quantify the specific contributions of each modality, an ablation study was conducted under a unified TCN architecture (table 5). Excluding STAT features led to a notable increase in MAE during rapid hemodynamic transitions, confirming STAT’s role in capturing high-frequency pressure dynamics. The removal of PTT-related features led to greater cumulative drift over long recordings, validating the use of timing features for baseline maintenance. Furthermore, omitting SQI and HR features significantly increased error variance in segments containing motion artifacts, demonstrating that including signal quality metrics is essential for the model to dynamically weight reliable sensor inputs. These results collectively justify the multi-modal fusion framework as a necessary approach for robust, real-time BP estimation.

**Table 5.** LOSOCV validation result of BP estimation vs Reference—Ablation study.

	Estimation error in mmHg (MAD)		
BP Estimation method	SBP [95% CI]	DBP [95% CI]	MBP [95% CI]
TCN model without STAT	7.14 [7.06, 7.21]	4.53 [4.49, 4.58]	5.08 [5.03, 5.13]
TCN model without PTT	6.28 [6.22, 6.34]	4.85 [4.80, 4.89]	4.96 [4.92, 5.00]
TCN model without HR/SQI	6.46 [6.39, 6.52]	4.00 [3.96, 4.04]	4.43 [4.39, 4.47]

Furthermore, adding calibration points every 10 or 20 min led to a further reduction in MAD for SBP, bringing it below 5 mmHg (Grade A). This improvement demonstrates the potential of incorporating time-based recalibration to enhance the accuracy of BP estimation. Going a step further, implementing a need-based calibration approach—triggered by signal quality changes or significant BP fluctuations—could offer even greater improvements, ensuring that recalibration is performed only when necessary, thereby optimizing the algorithm’s performance in real-time monitoring scenarios. This flexibility positions the TCN-based approach as a highly effective and adaptive solution for dynamic BP estimation.

A key advantage of the TCN method lies in its superior performance in detecting dynamic BP changes, which sets it apart from other approaches that primarily focus on static BP estimation. Many existing studies report BP values at isolated time points or during periods of rest, failing to account for BP fluctuations. This can result in lower reported MAD values, as the BP remains relatively stable. In our study, we used sustained handgrip exercise to introduce BP fluctuations, providing a more robust evaluation of model performance during dynamic BP phases. The results demonstrated that during stable periods, the PTT method achieved the best BP estimation; this is because the PTT method produced relatively stable results that did not reflect the actual BP variations. In periods of minimal BP fluctuation, the consistency between the stable BP and the PTT estimations created the illusion of better performance, stemming from its inherent lack of sensitivity to rapid hemodynamic changes. PTT relies on blood volume surrogates and vessel wall stiffness, which are heavily influenced by vasomotor tone and may not reflect acute beat-to-beat BP fluctuations accurately. In stable periods, where BP variation is minimal, a model that ‘drifts’ less or remains near the mean will naturally yield a lower MAD. However, clinical utility depends on the model’s sensitivity to BP swings. During the dynamic segment, PTT’s performance significantly degrades (MAD 8.57 mmHg), whereas the TCN model maintains robust accuracy (MAD 7.12 mmHg). The TCN model prioritizes dynamic sensitivity by leveraging STAT’s direct pressure morphology, which, while more complex to process, provides the high-fidelity information necessary to track real-time BP crises that PTT-only models would overlook. The TCN’s ability to effectively capture and respond to rapid BP changes highlights its distinct advantage in dynamic BP monitoring compared to both PTT and STAT methods.

The custom wearable device utilizes a specialized mechanical structure designed to apply an initial applanation force that flattens, but does not occlude, the STA. During exercise, while the physical hold-down pressure may inevitably fluctuate due to motion or physiological changes, our approach shifts the focus from ‘mechanical maintenance’ to ‘algorithmic compensation’. Specifically, we implemented a three-layer strategy to address hold-down pressure drift: (1) Real-time Tracking: A median filter with a 5 min window was employed to capture and track the slow, gradual drift of the baseline pressure signal. (2) Signal quality gating: An SQI, derived from template matching and ECG-STAT beat agreement, was used to identify and exclude segments where motion artifacts compromised the signal beyond the compensatory range of the model (SQI $ < $ 0.7). (3) Multi-modal fusion: The TCN integrates STAT morphology with ECG-derived HR and ECG-PPG-derived PTT. By fusing these heterogeneous signals, the model can cross-reference timing features (PTT) with morphological features (STAT), effectively ‘learning’ to decouple true BP variations from hold-down pressure artifacts.

Using MAD as the sole criterion for BP evaluation presents significant limitations. For instance, if we calculate the average BP values over a fluctuating period and use this fixed average to calculate the MAD at every point during that period, we would obtain artificially low MAD values. For example, by calculating the average SBP (as well as the average DBP and MBP) for each subject and comparing this fixed value with the actual BP values point by point along the SBP (as well as the DBP and MBP) curve, we found that the MAD values for this dataset were 4.95, 3.13, and 3.62 mmHg for SBP, DBP, and MBP, respectively. While this fixed estimation yields a lower MAD compared to each estimation algorithm, it completely fails to reflect the dynamic BP fluctuations. This illustrates the inadequacy of relying solely on MAD, as it can favor simplistic, static estimations that obscure critical real-time variations in BP.

We introduced the Pearson correlation coefficient (PCC) as a key metric for evaluating dynamic BP estimation, as it effectively measures how well an algorithm tracks real-time BP fluctuations. The PCC is calculated by comparing the BP values generated by the algorithm with those recorded by the Finapres reference. By using PCC, we can better assess the performance of different BP estimation methods. As shown in table 6, the TCN model achieved a PCC of 0.82 for SBP, 0.87 for DBP, and 0.85 for MBP, indicating a stronger correlation with the reference values than the STAT and PTT methods. These results highlight the superior ability of the TCN model to accurately capture dynamic BP changes, making it a more reliable choice for monitoring real-time BP fluctuations.

**Table 6.** Correlation index of the LOSOCV validation result between BP estimation and Finapres reference.

	PCC correlation index		
BP Estimation Method	SBP	DBP	MBP
STAT	0.74	0.77	0.73
PTT	0.79	0.76	0.75
TCN	0.82	0.87	0.85

Table 7 presents a comparison of our method with state-of-the-art noninvasive BP estimation algorithms. Many previous studies, particularly those utilizing deep learning models, reported lower MAD values. For instance, (Sadrawi *et al* 2020) achieved MAD values of 3.26 mmHg for SBP and 1.91 mmHg for DBP using a DCAE model, while El-Hajj and Kyriacou (2021) reported MAD values of 2.58 mmHg for SBP and 1.26 mmHg for DBP using an LSTM/GRU model. However, these studies employed a sample-wise validation approach, which risks data leakage. In this approach, samples from the same subject may be randomly split between the training and validation sets, leading to over-optimistic results. This method fails to truly test the model’s ability to generalize to new subjects. In contrast, our method utilizes subject-wise validation, ensuring that the training set does not contain any data or information related to the test subject. The training and testing data are completely independent, with no fine-tuning applied. This approach provides a more rigorous and realistic assessment of the model’s ability to generalize. Compared to other subject-wise validation studies, such as Baek *et al* (2019), our method demonstrated superior BP estimation performance. While Hill *et al* (2021) did not report MAD values for their V-Net model, they provided mean and standard deviation (STD) errors of $-$2.40 $\pm$ 5.62 mmHg for SBP and $-$2.50 $\pm$ 3.79 mmHg for DBP using subject-wise validation. In comparison, our method achieved better results, with a mean and STD of $-$0.85 $\pm$ 7.49 mmHg for SBP and $-$2.03 $\pm$ 5.32 mmHg for DBP. Although the databases used in these studies differ, and a direct comparison may not be entirely fair, our method consistently demonstrated stronger performance under subject-wise validation conditions, reinforcing its robustness and reliability in real-world applications.

**Table 7.** Performance comparison for BP estimation (Estimation error in mmHg (MAD)).

Authors	Algorithms	Validation	Dynamic BP testing	SBP	DBP
Ding *et al* (2015)	PTT-BP model	—	Yes	4.54	3.99
Sadrawi *et al* (2020)	DCAE	sample-wise	No	3.26 $\pm$ 0.31	1.91 $\pm$ 0.14
El-Hajj and Kyriacou (2021)	LSTM/GRU	sample-wise	No	2.58 $\pm$ 3.35	1.26 $\pm$ 1.63
Ibtehaz *et al* (2020)	U-Net	sample-wise	No	5.72 $\pm$ 9.16	3.45 $\pm$ 6.15
Kim *et al* (2022)	Self-attention	sample-wise	Yes	3.40 $\pm$ 4.36	1.75 $\pm$ 2.25
Ma *et al* (2024)	Transformer	sample-wise	No	3.37	2.48

Baek *et al* (2019)	CNN	subject-wise	No	9.30 $\pm$ 8.85	5.12 $\pm$ 5.52
Ours	TCN	subject-wise	Yes	5.58	4.34

Our results show that the multi-modal fusion significantly outperforms PTT-only baselines. This aligns with the view that tonometry (STAT) yields superior waveform quality compared to PPG for pressure estimation. The TCN effectively utilized the rich morphological features from the STAT waveform to track dynamic BP changes (induced by handgrip) that PTT often misses due to its reliance on vessel wall stiffness changes alone.

**Figure 9.**
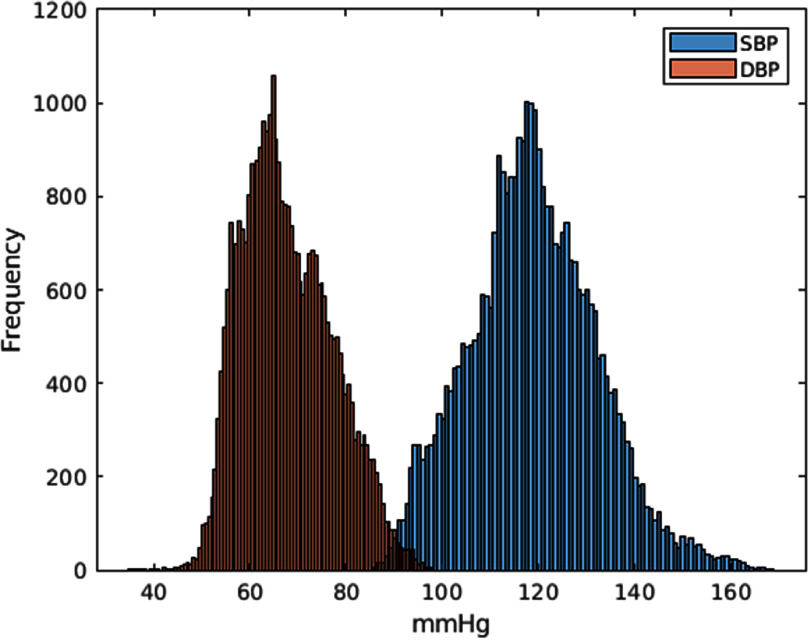
SBP and DBP distribution of our dataset.

This study has several limitations. First, the size of the training dataset was relatively small, which may affect the model’s generalizability. Second, all participants in the current cohort were healthy, normotensive volunteers, which limits the evaluation across broader BP phenotypes. Although isometric handgrip exercises successfully induced prominent, dynamic beat-to-beat BP fluctuations (as illustrated by the broad data distribution in figure 9), this cohort does not fully capture the altered vascular compliance and arterial remodeling characteristics inherent to chronic hypertension or hypotension. Consequently, validating this multi-modal fusion framework on diverse clinical populations with pathological BP variations represents an essential next step. Active, ongoing clinical data collection is underway to expand the dataset to include both hypertensive and hypotensive cohorts, which will allow for a more comprehensive assessment of the model’s robustness and clinical generalizability. Third, low-quality data were excluded from the analysis, potentially introducing a selection bias. Following the signal quality screening protocol, 20.9% of the total continuous recording data was excluded from the final analysis due to composite SQI values falling below the 0.7 threshold, primarily caused by severe motion artifacts or momentary loss of sensor contact during the physical perturbation phase. Crucially, because these transient quality dropouts were short-lived and non-overlapping across all modalities simultaneously, the TCN model’s dilated causal structure and multi-modal fusion strategy allowed it to bridge these short gaps, thereby ensuring that BP tracking remained robust and practically continuous throughout the monitoring sessions. Nevertheless, the use of LOSOCV allowed us to achieve promising results by mitigating the impact of limited data and ensuring robust performance evaluation. This demonstrates that our TCN model learns generalized physiological features rather than memorizing individual subject characteristics. As we continue to gather additional data from our hardware system in the lab and at MGH hospital, we expect that a larger dataset will significantly improve the model’s generalization capabilities and overall performance.

In conclusion, we developed a dynamic BP estimation algorithm based on multi-modality wearable, noninvasive signals using a deep learning approach. This method shows great potential for providing continuous, real-time BP estimation, and with a larger dataset, even better results are expected.

## Data Availability

The data cannot be made publicly available upon publication because no suitable repository exists for hosting data in this field of study. The data that support the findings of this study are available upon reasonable request from the authors.
